# Changes in executive functions and self-efficacy are independently associated with improved usual gait speed in older women

**DOI:** 10.1186/1471-2318-10-25

**Published:** 2010-05-19

**Authors:** Teresa Liu-Ambrose, Jennifer C Davis, Lindsay S Nagamatsu, Chun Liang Hsu, Lindsay A Katarynych, Karim M Khan

**Affiliations:** 1 Centre for Hip Health and Mobility, Vancouver Coastal Health Research Institute, University of British Columbia, Vancouver, BC, Canada; 2 Department of Physical Therapy, University of British Columbia, Vancouver, BC, Canada; 3 Department of Psychology, University of British Columbia, Vancouver, BC, Canada; 4 Department of Family Practice, University of British Columbia, Vancouver, BC, Canada

## Abstract

**Background:**

Improved usual gait speed predicts substantial reduction in mortality. A better understanding of the modifiable factors that are independently associated with improved gait speed would ensure that intervention strategies are developed based on a valid theoretical framework. Thus, we examined the independent association of change in executive functions and change in falls-related self-efficacy with improved gait speed among community-dwelling senior women.

**Methods:**

A secondary analysis of the 135 senior women aged 65 to 75 years old who completed a 12-month randomized controlled trial of resistance training. Usual gait speed was assessed using a 4-meter walk. Three executive processes were assessed by standard neuropsychological tests: 1) set shifting; 2) working memory; and 3) selective attention and response inhibition. A linear regression model was constructed to determine the independent association of change in executive functions and falls-related self-efficacy with change in gait speed.

**Results:**

Improved selective attention and conflict resolution, and falls-related self-efficacy, were independently associated with improved gait speed after accounting for age, global cognition, baseline gait speed, and change in quadriceps strength. The total variance explained was 24%.

**Conclusions:**

Interventions that target executive functions and falls-related self-efficacy, in addition to physical functions, to improve gait speed may be more efficacious than those that do not.

**Trial Registration:**

ClinicalTrials.gov Identifier: NCT00426881

## Background

Gait speed is a global indicator of functional mobility and status. Reduced speed is associated with risk of major health-related outcomes [1], including falls [2] and fracture [3], in older adults. It is also associated with sub-clinical structural brain abnormalities [4] and cognitive function [5]. Improved usual gait speed over a 12-month period predicts substantial reduction in mortality [6].

Although there has been a call for research to determine whether interventions to improve gait speed affect survival [6], a better understanding of the modifiable factors that are independently associated with improved gait speed is needed. Candidates include cognitive function, self-efficacy, and physiological function, such as muscular strength. A better understanding of these key factors would ensure that intervention strategies to improve gait speed are based on a valid theoretical framework.

Current evidence suggests that executive functions should be considered in interventions to improve gait speed in older adults. The Health, Aging and Body Composition Study [7] highlighted that baseline lower executive functions predict subsequent decline in gait speed. Conversely, prospective studies demonstrate that baseline gait speed predicts subsequent cognitive decline [8]. Together, evidence supports the hypothesis that cognitive and physical functions are inter-related.

Executive functions are higher order cognitive processes that control, integrate, organize, and maintain other cognitive abilities [9]. These cognitive processes include the ability to concentrate, to attend selectively, and to plan and to strategize. Executive functions decline substantially with aging [10] as does the corresponding volume of the frontal-subcortical neuronal system [9,11-13]. Importantly, reduced executive functioning is prevalent even among healthy, community-dwelling seniors with intact global cognitive function [14,15]. This is not surprising given many of the pathological changes (e.g., white matter lesions, reduced frontal-subcortical volume) associated with reduced executive functions are prevalent but clinically silent [16].

Evidence from neuro-imaging studies provides insight to possible mechanisms underlying the association between executive functions and gait speed. Specifically, cerebral white matter lesions -- which are associated with reduced executive functioning [16] - are also associated with gait and balance abnormalities [17,18]. Cerebral white matter lesions may interrupt frontal lobe circuits responsible for normal gait and balance or they may interfere with long loop reflexes mediated by deep white matter sensory and motor tracts [19]. In addition, the periventricular and subcortical distribution of white matter lesions could interrupt the descending motor fibers arising from medial cortical areas, which are important for lower extremity motor control [20].

Falls-related self-efficacy is associated with gait performance [21,22] and hence, should also be considered in interventions to improve gait speed. Based on Bandura's Social Cognitive Theory [23], which states that perceived capability predicts activity better than does actual physical ability, improved self-efficacy should be related to improved gait speed. Using bivariate correlation analysis, we previously demonstrated that change in falls-related self-efficacy was not significantly associated with change in gait speed [24]. However, we did not consider executive functions in these models -- these cognitive processes may play a significant role in older adults' ability to accurately perceive their actual physical capacity [25].

To our knowledge, it is currently unknown whether *change *in executive functions and falls-related self-efficacy are independently associated with change in gait speed in older adults after accounting for relevant factors such as age and global cognitive function. Yet, such knowledge would facilitate the development and refinement of targeted interventions to improve gait speed in older adults. For example, if improved executive functioning were independently associated with improved gait speed, it would be logical to incorporate cognitive training that focused specifically on executive functioning rather than other domains of cognition such as memory. Thus, we examined the independent association of change in three key executive processes and change in falls-related self-efficacy with improved gait speed among community-dwelling senior women after accounting for age, global cognition, baseline gait speed, and change in quadriceps strength.

## Methods

### Study Design and Participants

The sample for this analysis consisted of 135 women who consented and completed a 12-month randomized controlled trial of exercise that aimed to examine the effect of once-weekly or twice-weekly resistance training compared with a twice-weekly balance and tone exercise intervention on cognitive performance of executive functions. The design and the primary results of the study have been reported elsewhere [26].

We recruited women who: 1) were aged 65 to 75 years; 2) were living independently in their own home; 3) scored > 24 on the Mini-Mental State Examination (MMSE); and 4) had a visual acuity of at least 20/40, with or without corrective lenses. Recruitment strategies have been described previously [26]. Ethical approval was obtained from the Vancouver Coastal Health Research Institute (V06-0326) and the University of British Columbia's Clinical Research Ethics Board (H06-0326). All participants provided written informed consent.

### Exercise Intervention

#### Resistance Training

All classes were 60 minutes in duration. The protocol for this program was progressive and high-intensity in nature. Both a Keiser^® ^Pressurized Air system and free weights were used to provide the training stimulus. Other key strength exercises included mini-squats, mini-lunges, and lunge walks.

#### Balance and Tone

This program consisted of stretching exercises, range of motion exercises, kegals, balance exercises, and relaxation techniques. This group served to control for confounding variables such as physical training received by traveling to the training centres, social interaction, and lifestyle changes secondary to study participation.

### Descriptive Variables

Global cognition was assessed using the MMSE [27]. We used the 15-item Geriatric Depression Scale (GDS) [28] to screen for depression. Functional Comorbidity Index was calculated to estimate the degree of comorbidity associated with physical functioning [29]. This scale's score is the total number of comorbidities.

### Dependent Variable: Gait Speed

Participants were asked to walk at their usual pace along a 4-meter path. Gait speed (m/s) was calculated from the mean of two trials. The test-retest reliability of gait speed in our laboratory is 0.95 (ICC) [21].

### Independent Variables of Interest

#### Dominant Quadriceps Strength

A simple strain gauge was used to assess dominant quadriceps (isometric) strength to the nearest 0.5 kilogram [30]. Participants were seated with the hip and the knee joint at 90 degrees of flexion. The best of three trials was recorded.

#### Cognitive Performance of Executive Processes

For this study, we referred to the work by Miyake and colleagues [31] who identified three key executive processes that are moderately correlated with one another but each has a distinct purpose; they are: 1) set shifting; 2) working memory; and 3) selective attention and conflict resolution.

##### Set Shifting

We used the Trail Making Tests (Part A & B) to assess set shifting [32]. Part A assesses psychomotor speed and requires the participant to draw lines that connect encircled numbers sequentially. Part B consists of encircled numbers and letters. Participants were instructed to draw a line as quickly and as accurately as possible from 1 to A, A to 2, 2 to B, B to 3, and so on, until they completed the task. We recorded the amount of time (in seconds) it took to complete each task. To index set shifting, we calculated the difference between Part B and Part A completion time. Smaller difference scores indicate better set shifting.

##### Working Memory

We used the verbal digits forward and verbal digits backward tests to index the central executive component of working memory [32]. Both tests consist of seven pairs of random number sequences that the assessor reads aloud at the rate of one per second. The sequence begins with three digits and increases by one at a time up to a length of nine digits. The test includes two sequences of each length and testing ceases when the participant fails to recollect any two with the same length. The score recorded, ranging from 0 to 14, is the number of successful sequences. For the verbal digits forward test, the participant's task is to repeat each sequence exactly as it is given. For the verbal digits backward test, the participant's task is to repeat each sequence in the reversed order. The difference between the verbal digits forward test score and the verbal digits backward test score was used as an index of the central executive component of working memory. Smaller difference scores indicate better working memory.

##### Selective Attention and Conflict Resolution

We used Graf, Uttl and Tuokko's [33] version of the Stroop Test to assess selective attention and conflict resolution. For the Stroop Test, there were three conditions. First, participants were instructed to read out words printed in black ink (e.g., BLUE). Second, they were instructed to read out the colour of coloured-X's. Finally, they were shown a page with colour-words printed in incongruent coloured inks (e.g., the word "BLUE" printed in red ink). Participants were asked to name the ink colour in which the words are printed (while ignoring the word itself). There were 80 trials for each condition and we recorded the time participants took to read each condition. The ability to selectively attend and control response output was calculated as the time difference between the third condition and the second condition. Smaller time differences indicate better selective attention and conflict resolution.

#### Falls-Related Self-Efficacy

The 16-item Activities-specific Balance Confidence (ABC) Scale [34] assessed fall-related self-efficacy with each item rated from 0% (no confidence) to 100% (complete confidence); it provides a score out of 100%.

### Data Analyses

Descriptive data are reported for variables of interest. Data were analyzed using SPSS Windows Version 17.0 (SPSS Inc., Chicago, IL). The associations between the variables were determined using the Pearson product moment coefficient of correlation.

A multiple linear regression model was constructed to determine the independent association of change in executive processes and change in falls-related self-efficacy with change in gait speed over a 12-month period. Change in gait speed, falls-related self-efficacy, and dominant quadriceps strength was calculated by subtracting the baseline value from the trial completion value. Change in the three executive functions was calculated by subtracting the trial completion value from the baseline value. Experimental group, age, global cognition, baseline gait speed, and the change in dominant quadriceps strength were statistically controlled by entering these four variables into the regression model first. These independent variables were determined from the results of the Pearson product moment coefficient of correlation analyses and based on biological relevance, such as experimental group, baseline global cognition, and change in dominant quadriceps strength.

The three executive processes were then entered into regression model and only those that significantly improved the model were kept in the model (i.e., significant Rsq change). The ABC Scale score was entered last into each model. Alpha was set at ***P ***< 0.05.

## Results

### Participants and Change in Variables of Interest

Of the 155 participants who consented and were randomized at baseline, 135 completed the 12-month exercise trial. Table 1 reports the baseline descriptive statistics for this cohort.

**Table 1:** Descriptive statistics for variables of interest at baseline.

Variable *	BAT (n = 42) Mean (SD)	1× RT (n = 47) Mean (SD)	2× RT (n = 46) Mean (SD)	Total (N = 135) Mean (SD)
Age (yr)	69.9 (3.0)	69.5 (2.6)	69.4 (3.0)	69.6 (2.9)
Height (cm)	161.3 (6.9)	161.7 (6.9)	162.2 (6.2)	161.7 (6.6)
Weight (kg)	67.9 (11.0)	70.7 (15.5)	70.0 (14.6)	69.6 (13.9)
Education				
Less than Grade 9 ^†^	1.0 (2)	1.0 (1.9)	1.0 (1.9)	3.0 (1.9)
Grade 9 to 12 without Certificate or Diploma ^†^	2.0 (4.1)	3.0 (5.6)	4.0 (7.7)	9.0 (5.8)
High School Certificate or Diploma ^†^	6.0 (12.2)	9.0 (16.7)	10.0 (19.2)	25.0 (16.1)
Trades or Professional Certificate or Diploma ^†^	14.0 (28.6)	10.0 (18.5)	6.0 (11.5)	30.0 (19.4)
University Certificate or Diploma ^†^	7.0 (14.3)	12.0 (22.2)	9.0 (17.3)	28.0 (18.1)
University Degree ^†^	19.0 (38.8)	19.0 (35.2)	22.0 (42.3)	60.0 (38.7)
MMSE Score (max. 30 pts)	28.9 (1.2)	28.5 (1.3)	28.5 (1.5)	28.6 (1.3)
Falls in the Last 12 Months (yes/no) ^†^	16 (34)	13 (24.5)	20 (38.5)	49 (32.2)
Geriatric Depression Scale (max. 15 pts)	0.5 (1.9)	0.3 (1.0)	0.9 (2.4)	0.6 (1.8)
Functional Comorbidity Index (max. 18 pts)	2.2 (1.7)	1.8 (1.6)	2.2 (1.5)	2.1 (1.6)
Lawton and Brody (max. 8 pts)	8.0 (0)	8.0 (0.1)	7.9 (0.5)	8.0 (0.3)
Dominant Quadriceps Strength (kg)	27.9 (8.0)	30.4 (6.7)	29.8 (8.3)	29.4 (7.7)
Stroop CW - Stroop C (sec)	44.03 (15.4)	45.71 (24.8)	46.07 (16.0)	45.31 (19.2)
Trail B - Trail A (sec)	44.60 (36.9)	41.36 (26.2)	49.12 (37.7)	45.01 (33.8)
Digit Forward - Digit Backward	3.4 (2.6)	3.4 (2.0)	3.4 (2.4)	3.4 (2.3)
Gait Speed (m/sec)	1.16 (0.2)	1.18 (0.2)	1.16 (0.2)	1.17 (0.2)
ABC Scale Score (%)	85.3 (15.0)	89.4 (12.1)	89.5 (10.7)	88.2 (12.7)

* BAT = Balance and Tone; 1× RT = once-weekly resistance training; 2 × RT = twice-weekly resistance training; yr = year; kg = kilogram; MMSE = Mini-Mental State Examination; sec = seconds; Stroop CW = Stroop colour-words condition; Stroop C = Stroop coloured-X's condition; m/sec = meters per second; ABC Scale = Activities-specific Balance Confidence.

^† ^Count = number of "yes" cases within each group. % = percent of "yes" within each group.

At the end of the 12-month trial, the 135 women demonstrated a mean increase of 7 kilograms in dominant quadriceps strength and a four- and nine-second reduction in Stroop Test and Trails Making Tests completion time, respectively (Table 2). There was a 1.2% increase in the ABC Scale score. Usual gait speed increased by a mean of 0.22 m/s. A paired t-test indicated that this was a statistically significant increase (***P ***< 0.001).

**Table 2:** Mean change for variables of interest.

Variable *	BAT (n = 42) Mean (SD)	1× RT (n = 47) Mean (SD)	2 × RT (n = 46) Mean (SD)	Total (N = 135) Mean (SD)
Δ Dominant Quadriceps Strength (kg) **	7.1 (7.0)	6.4 (9.1)	8.1 (6.4)	7.2 (7.6)
Δ Stroop CW - Stroop C (sec) ^†^	0.26 (17.12)	6.22 (22.31)	5.01 (13.75)	3.94 (18.21)
Δ Trail B - Trail A (sec) ^†^	8.64 (32.15)	7.30 (30.36)	10.96 (36.92)	8.97 (33.06)
Δ Digit Forward - Digit Backward ^†^	-0.64 (2.70)	0.06 (2.54)	-0.47 (2.24)	-0.34 (2.50)
Δ Gait Speed (m/sec) **	0.22 (0.18)	0.19 (0.19)	0.24 (0.16)	0.22 (0.18)
Δ ABC Scale Score (%) **	2.83 (9.91)	2.26 (10.27)	-1.37 (10.60)	1.19 (10.37)

* BAT = Balance and Tone; 1× RT = once-weekly resistance training; 2 × RT = twice-weekly resistance training; Stroop CW = Stroop colour-words condition; Stroop C = Stroop coloured-X's condition; m/sec = meters per second; ABC = Activities-Specific Balance Confidence.

** Mean change = final value minus baseline value. Positive change indicates improvement.

^† ^Mean change = baseline value minus final value. Positive change indicates improvement.

### Correlation Coefficients

Table 3 reports the correlation coefficients of those variables included in the final multi-variable regression model. Of the three key executive processes only change in selective attention and conflict resolution was significantly and positively associated with improved gait speed (*P *< 0.01). Change in falls-related self-efficacy was also significantly and positively associated with improved gait speed (*P *< 0.001).

**Table 3:** Multiple linear regression model summary improved usual gait speed.

	Δ Usual Gait Speed (m/s)					
	
Independent Variable	r	R^2^	R^2 ^Change	Unstandardized B (Standard Error)	Standardized *β*	***P ***- value
**Model 1**	0.345	0.119	0.119			
Group	0.082			0.014 (0.018)	0.066	0.441
Age	-0.19 *			-0.014 (0.005)	-0.239	0.007
MMSE Score	0.02			0.004 (0.011)	0.028	0.749
Baseline Gait Speed	-0.19 *			-0.249 (0.081)	-0.273	0.003
Δ Quadriceps Strength	0.11			0.003 (0.002)	0.129	0.133
**Model 2**	0.441	0.194	0.075			
Group	0.082			0.008 (0.018)	0.036	0.659
Age	-0.19 *			-0.013 (0.005)	-0.217	0.011
MMSE Score	0.02			0.002 (0.011)	0.015	0.859
Baseline Gait Speed	-0.19 *			-0.283 (0.078)	-0.310	< 0.001
Δ Quadriceps Strength	0.11			0.004 (0.002)	0.173	0.038
Δ Stroop Test	0.26 *			0.003 (0.001)	0.283	0.001
**Model 3**	0.490	0.240	0.045			
Group	0.082			0.017 (0.018)	0.077	0.349
Age	-0.19 *			-0.014 (0.005)	-0.236	0.005
MMSE Score	0.02			0.001 (0.011)	0.008	0.922
Baseline Gait Speed	-0.19 *			-0.248 (0.077)	-0.271	0.002
Δ Quad Strength	0.11			0.002 (0.002)	0.086	0.324
Δ Stroop Test	0.26 **			0.002 (0.001)	0.229	0.007
Δ ABC Scale	0.29 ***			0.004 (0.002)	0.236	0.008

* ***P ***≤ 0.05

** ***P ***≤ 0.01

*** ***P ***≤ 0.001

### Linear Regression Model

Experimental group, age, baseline gait speed, baseline global cognition, and change in dominant quadriceps strength accounted for 11.9% of the variance in improved gait speed (Table 3). Adding the change score for the executive process of selective attention and conflict resolution to the model resulted in an R-square change of 7.5% and significantly improved the regression model (*F *Change = 11.6, ***P ***= 0.001). Adding change in the ABC Scale score to the model resulted in an R-square change of 4.5% and significantly improved the model (*F *Change = 7.4, *P* = 0.008). The total variance accounted by the final model was 24.0% (Table 3).

## Discussion

There is an increasing recognition that gait speed is an important indicator of current and future functional status in older adults. We found that both improved cognitive performance of selective attention and conflict resolution and falls-related self-efficacy were independently associated with improved gait speed among community-dwelling senior women. To our knowledge, this is the first study that has examined the independent association of changes in executive functions and falls-related self-efficacy with improved gait speed after accounting for age global cognition, baseline gait speed, and change in dominant quadriceps strength.

Our findings concur with previous studies demonstrating the association between executive functions and gait speed [7,35]. Of the three key executive processes examined in this study, only improved performance on the Stroop Test was independently associated with improved gait speed after accounting for relevant covariates. This finding concurs with those of a previous cross-sectional study of community-dwelling older adults [36]. That study found that performance on the Stroop Test, but not on the Trail Making Test (Part A & B), was independently associated with usual gait speed. However, our results contradict those of the Lifestyle Interventions and Independence for Elders Pilot (LIFE-P) study [37]; that study found that improved performance on the Stroop Test was associated with improved balance but not with improved gait speed. Differences in study participants and interventions may contribute to this discrepancy. It is not surprising that global cognition was not significantly associated with increased gait speed in our study given that only individuals without cognitive impairment were included.

As gait speed is an independent predictor of falls [2] and fracture risk [3], our finding extends previous studies demonstrating an association between the Stroop Test and falls [38,39]. Specifically, in a prospective study of inpatient falls in an urban rehabilitation hospital, performance on the Stroop Test predicted falls status beyond that explained by age and functional motor ability [38]. Neuro-imaging data also suggest that the specific executive cognitive process of selective attention and conflict resolution is associated with falls and mobility. Specifically, our previous work [40] found that senior fallers had reduced brain activation in the right cerebellum during the flanker task - a task requiring selective attention and conflict resolution -- compared with senior non-fallers. This alteration in neural activity may be an early indicator of increased falls risk as the cerebellum is not only responsible for normal motor function (i.e., movement), it is also involved in executive functions [41] (e.g., planning of movement), and hence, may represent a critical interface between mobility and cognition.

Our finding of an independent association between improved falls-related self-efficacy and improved gait speed extend those of two previous cross-sectional investigations [21,22]. Specifically, we demonstrated that falls-related self-efficacy was independently associated with gait speed among senior women after accounting for age, physical activity level, and relevant physiological functions [21]. We note that the observed change in falls-related self-efficacy was minimal in this study. This suggests that small changes in falls-related self-efficacy, as measured by the ABC Scale, are clinically significant. However, more research is needed to ascertain what constitutes a clinically significant change in the ABC Scale score among high-functioning community-dwelling older adults. The minimal change observed may also be secondary to a ceiling effect of the ABC Scale [42] in our study cohort.

According to the LIFE-P study [43], the observed improvement in gait speed of 0.22 m/s is a meaningful change. Specifically, that study reported a 0.08 m/s change in 4-meter gait speed to be substantial, with an effect size of at least 0.5. However, participants of the LIFE-P study were likely more frail than our study participants because the LIFE-P study participants were aged 70 to 89 years and had Short Physical Performance Battery scores of 9 or less. Therefore, the substantial change in gait speed we observed may be, at least in part, due to a greater capacity of our participants to improve.

Our finding that improved dominant quadriceps strength was not significantly associated with improved gait speed concurs with previous findings [44]. A model assuming nonlinear relationships may be appropriate for understanding how change in muscular strength affects change in gait speed [44].

An important clinical implication of these results is that clinicians may need to consider the specific executive process of selective attention and conflict resolution and falls-related self-efficacy in the rehabilitation of gait among community-dwelling seniors. Our data suggest interventions that target executive functions and falls-related self-efficacy in addition to physical functions to improve gait speed may be more efficacious than those that do not.

We note that our study sample consisted exclusively of independent community-dwelling senior women who were without significant physical and cognitive impairments and without a significant history of falls. Thus, the results of our study may not generalize beyond this population of senior women and we may have underestimated the contribution of change in executive functions and falls-related self-efficacy to change in gait speed. Future prospective studies are needed to test whether the present findings also apply to larger, more heterogeneous populations. Finally, because the effect of selective attention and conflict resolution and falls-related self-efficacy observed in this study was small, future studies are also needed to determine the clinical significance, if any, of the additional and unique variance accounted for by each factor.

## Conclusions

In conclusion, both change in executive functions and falls-related self-efficacy are independently associated with improved gait speed. Clinicians may need to consider these factors when developing rehabilitation strategies to improve gait speed.

## Competing interests

The authors declare that they have no competing interests.

## Authors' contributions

TLA conceived and designed the study, acquired data, and analyzed and interpreted the data. TLA, JCD, and KMK drafted and revised the manuscript. LSN, LK, and CLH acquired data and participated in the statistical analysis. All authors read and approved the final manuscript.

## Pre-publication history

The pre-publication history for this paper can be accessed here:

http://www.biomedcentral.com/1471-2318/10/25/prepub
